# Predictors of health-related quality-of-life after cardiac surgery: findings from the ANesthesiology-QUality-Registry (ANQUR) and frailty-management

**DOI:** 10.1186/s12871-026-03931-8

**Published:** 2026-05-25

**Authors:** Max Briefs, Janis Fliegenschmidt, Jan Wiesemann, Catharina Middeke, Astrid Bergmann, René Schramm, Nikolai Hulde, Jan Gummert, Vera von Dossow

**Affiliations:** 1 Ruhr University Bochum, Heart and Diabetes Center NRW, Institute of Anesthesiology and Pain Therapy, Georgstraße 11, 32545 Bad Oeynhausen, Germany; 2 Department of Anesthesiology, Intensive Care and Pain Therapy, Ruhr University Bochum, University Hospital Knappschaftskrankenhaus Bochum, In der Schornau 23- 25, Bochum, 44892 Germany; 3 Heart and Diabetes Center NRW, Department of Thoracic and Cardiovascular Surgery, Ruhr University Bochum, Georgstraße 11, Bad Oeynhausen, 32545 Germany

**Keywords:** Cardiac surgical procedures, Quality of life, Follow-up studies, Patient reported outcome measures, Frailty, Cognitive dysfunction

## Abstract

**Background:**

Assessment of long-term patient-reported outcome allows identification of vulnerable populations undergoing cardiac surgery. Incorporation of findings into clinical practice may enhance risk-stratification, prevent perioperative complications, and improve outcome. Frailty is prevalent in up to a third of cardiac patients and peri-procedural programs addressing frailty and delirium may be relevant to long-term quality-of-life. This prospective study aims to identify predictors of health-related quality-of-life (HR-QoL) one year after cardiac surgery and assess outcome in patients enrolled in an extensive frailty- and delirium-pathway.

**Methods:**

Patients undergoing cardiac surgery at a high-volume German heart center were enrolled in an anesthesiology quality registry and health-related quality of life was assessed using the Short Form-12 (SF-12) questionnaire at 1-year-follow-up. Corresponding factors were analyzed for their association with individual outcome. The cohort comprised 812 patients. A subgroup of 190 patients participated in a frailty and delirium management program, providing extended preoperative screening and postoperative supervision.

**Results:**

Female sex [B -2.59, 95% CI (-4.39 – -0.78), *p* = 0.005], increase in age [B -3.1, 95% CI (-5.34 – -0.86), *p* = 0.007] and weight [B -3.97, 95% CI (-6.03 – -1.91), *p* < 0.001], preoperative anemia [B -3.21, 95% CI (-5.64 – -0.77), *p* = 0.01], history of smoking [B -3.0, 95% CI (-5.39 – -0.6), *p* = 0.014], failure to extubate after 8 hours of postoperative ventilation [B -1.82, 95% CI (-3.36 – -0.28), *p* = 0.021], postoperative acute kidney injury [B -2.18 (95% CI -4.22 – -0.14), *p* = 0.037] and physical frailty [B -9.88, 95% CI (-18.28 – -1.49), *p* = 0.021] were independently associated with lower physical outcome scores. Cognitive scores were higher in older patients [B 4.08, 95% CI (1.57–6.59), *p* = 0.001] and lower in smokers [B -2.43, 95% CI (-4.34 – -0.51), *p* = 0.013].

**Conclusion:**

Independent predictors of impaired HR-QoL at 1-year follow-up could be identified, suggesting a phenotype at risk. Physical frailty independently predicted poorer physical outcome, emphasizing potential for prehabilitation and frailty-management. Findings should be interpreted considering selection- and response-bias and absence of baseline HR-QoL-assessment.

**Trial registration:**

This observational study complied with the Declaration of Helsinki and was approved by the Ethics Committee of the Medical Faculty of the Ruhr-University Bochum on 17th November 2022 (Registration-Number: 2022 − 947). Minor additions to the questionnaire were approved on 19th August 2024 (Registration-Number: 2022 − 947_1).

**Graphical Abstract:**

**Figure:**
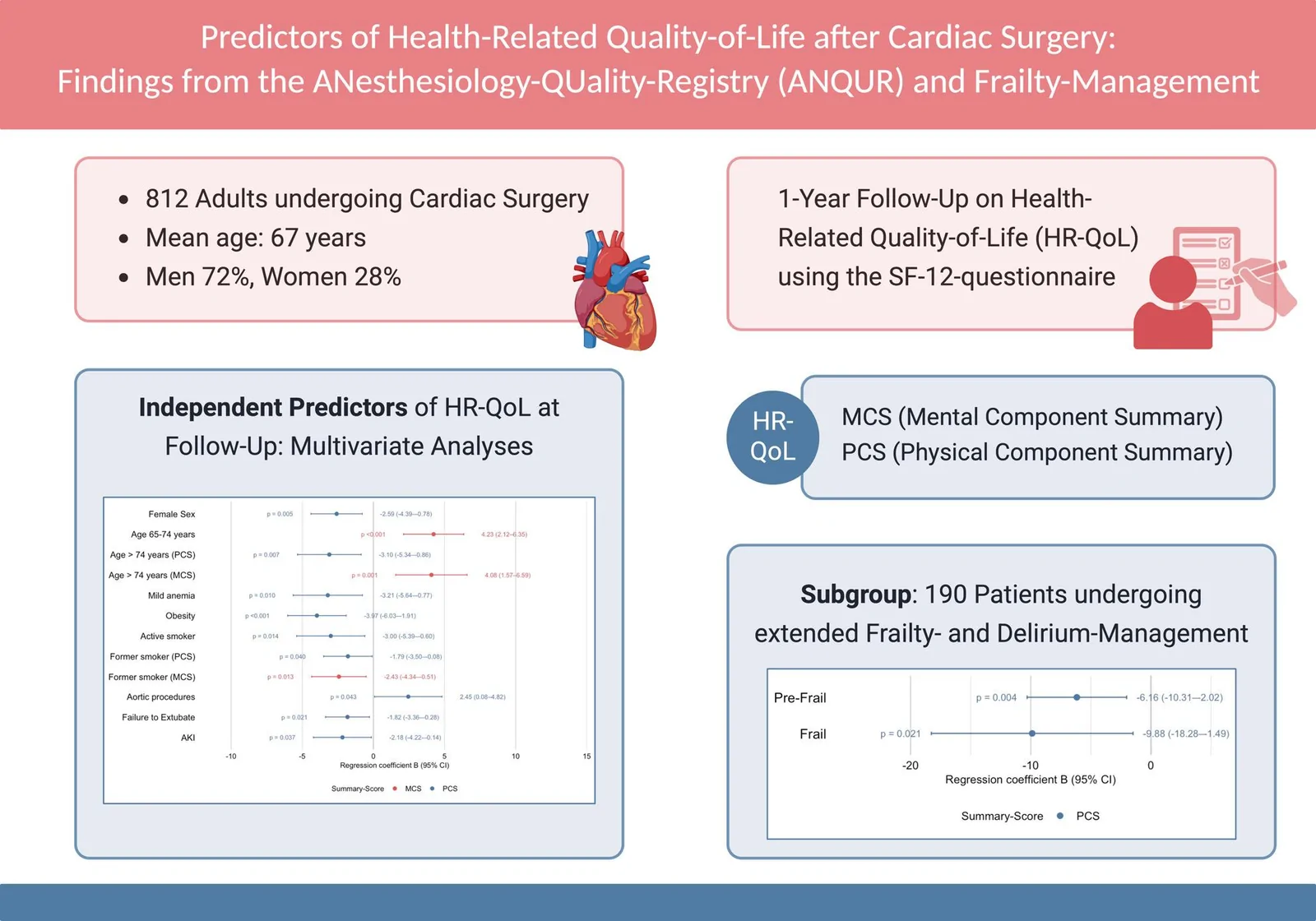

**Supplementary Information:**

The online version contains supplementary material available at 10.1186/s12871-026-03931-8.

## Background

In light of an ongoing demographic shift, hospitalized populations become older and increasingly comorbid, resulting in an incline in economic burden [1–4]. To minimize perioperative complications and financial downsides thereof, extensive risk-stratification is warranted [5]. In addition to short-term physician reported follow-up, patient-reported outcome (PRO) and health-related quality of life (HR-QoL) are increasingly recognized for evaluation of subjective long-term outcome [6, 7]. HR-QoL represents the most frequently assessed PRO and can be evaluated using the generic Short-Form-12-questionnaire (SF-12), which operationalizes QoL and yields both a physical and a mental component summary score [8–11]. The SF-12 user manual was first published in 1994, and the instrument has been validated in German, and standardized to the general population [10–12]. In addition to QoL-assessment, incorporation of findings into clinical practice is crucial and may enable identification of vulnerable populations, who might benefit from extended supervision and periprocedural care. Potentially modifiable predictors of impaired HR-QoL in patients undergoing cardiac surgery – such as weight, a history of smoking or length of stay – have been previously identified, though a lack of consistency across studies was apparent [13]. 

Prehabilitation represents a structured effort for preoperative modification of predictors of impaired outcome and aims to improve patients’ resilience and postoperative outcome by optimizing individual risk profile and thereby reducing overall complications, their severity and limiting further decline [14, 15]. One exemplary pillar of preoperative optimization is patient-blood-management (PBM); PBM can be defined as a patient-centered approach to improve outcomes and reduce risks while promoting patient safety and empowerment [16, 17]. Furthermore, incorporating multimodal concepts of nutrition and exercise can benefit both surgical and subjective outcomes. These interventions – applied not only in prehabilitation prior to surgery but also in rehabilitation or recovery after surgery (ERAS) – include specific strategies such as physical activation or guidance, nutritional counseling, and optimization, representing only a subset of potential targets for improving patient centered care [18, 19]. 

Frailty is described as a concept of age-related decline in physical function accompanied by increased vulnerability to external stressors and is prevalent in over 30% of patients undergoing cardiac surgery [20, 21]. The association between frailty, coexisting comorbidities, and increased odds of impaired outcomes in cardiac patients suggests that preoperative modification of frailty may represent a potential strategy to improve outcomes and prevent complications [21, 22]. This prospective study aims to identify determinants of clinical outcomes in patients undergoing cardiac surgery, while also focusing on prognostic significance of physical and cognitive frailty.

## Methods

### Objective and endpoints

The primary objective of this prospective observational study was to identify demographic, preoperative, intraoperative, and postoperative factors associated with health-related quality of life (HR-QoL) one year after cardiac surgery, assessed using the Short Form-12 (SF-12) questionnaire. A secondary objective was to evaluate whether physical and cognitive frailty, assessed in a dedicated frailty- and delirium-management subgroup, were independently associated with postoperative HR-QoL.

The primary endpoints were the individual Physical Component Summary (PCS) and Mental Component Summary (MCS) scores derived from the SF-12 questionnaire at 1-year follow-up. These scores were calculated according to the standardized scoring manual and are norm-based measures validated against the general population, with a mean of 50 and a standard deviation of 10; lower scores indicating poorer HR-QoL. The PCS and MCS scores provide a differentiated assessment of physical and mental health status and allow comparison both between patient groups and with the general population.

### Patient recruitment

**Fig. 1:**
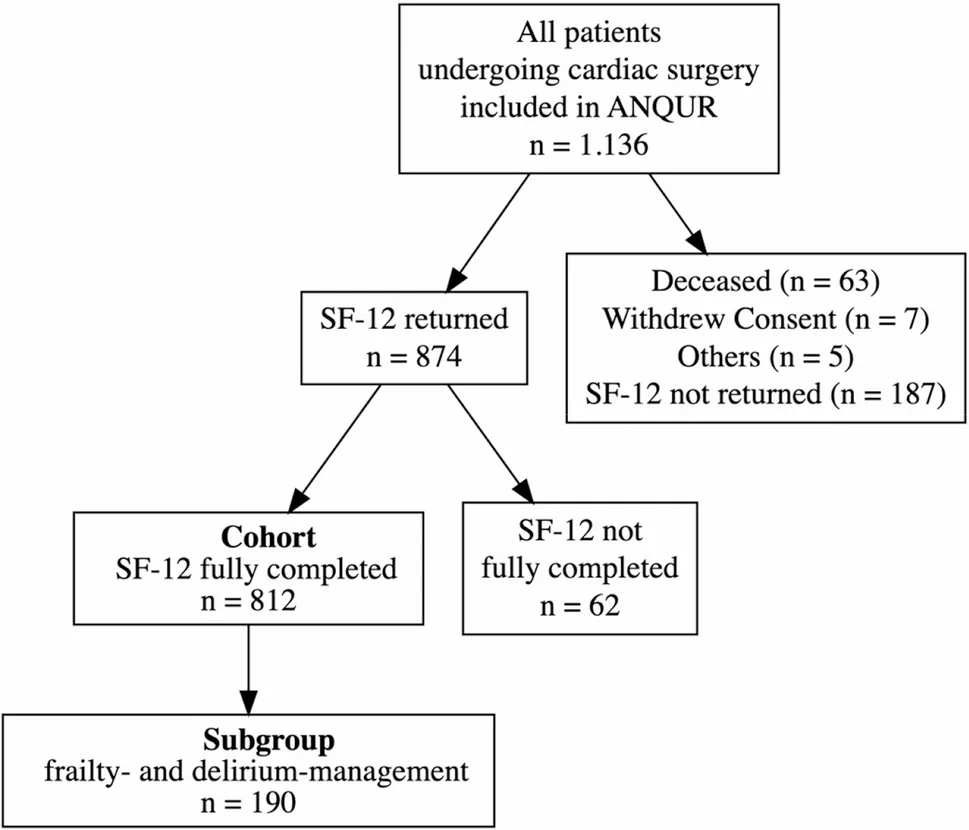
Flowchart of patient-selection (ANQUR: ANesthesiology-QUality-Registry, SF-12: Short-Form-12)

All consecutive adult patients undergoing cardiac surgery at a high-volume German heart center between January and December 2023 who provided written informed consent were eligible for inclusion in the registry. No formal a priori sample size calculation was performed due to the exploratory observational design; all consecutive eligible patients during the study period were included. In total, 1.136 patients who underwent cardiac surgery gave informed consent to participation in an Anesthesiology-Quality-Registry (ANQUR) in 2023. Patients were preoperatively informed and consent was recorded during the premedication consultation. Postoperatively, HR-QoL-data was assessed at 1-year-follow up, using the Short-Form-12-questionnaire (SF-12). Due to limited resources and logistic challenges, a preoperative assessment was not performed. The final cohort comprised 812 patients. A subgroup comprised 190 patients, who were additionally included in a specialized frailty- and delirium-pathway and underwent extensive preoperative screening for physical or cognitive frailty as well as assessment of risk for delirium (Fig. 1).

### Data collection and definition of variables

All available SF-12 data were included in the registry, and standardized scoring was performed for fully completed questionnaires. In accordance with the user manual, the Physical Component Summary (PCS) and Mental Component Summary (MCS) scores as endpoint variables were calculated for each patient, representing individual HR-QoL. The investigated variables comprised demographic and periprocedural factors, as well as parameters assessed within the frailty management program. All data were extracted from internal databases and pseudonymized prior to analysis. For analytical purposes, non-continuous variables were categorized as appropriate.

Missing data were not imputed due to their overall low proportion (see Table 1). Lipoprotein(a) was the only variable with a relatively high proportion of missing values (14.2 %), reflecting that it is not part of routine laboratory testing; in patients without recorded values, it is unlikely to have clinical relevance.

Demographic variables included sex and age. Sex was recorded as male or female, with no additional categories available. Age was analyzed as a continuous variable and additionally categorized into three predefined groups: 18–64, 65–74, and ≥ 75 years. Preoperative variables included hemoglobin levels, analyzed both as a continuous variable and categorized according to the World Health Organization (WHO) classification of anemia; LDL cholesterol and lipoprotein(a) [Lp(a)] levels as indicators of lipid profile; body mass index (BMI), assessed continuously and categorically; preexisting conditions including hypertension, diabetes mellitus, and peripheral arterial disease (PAD); smoking status; and left ventricular ejection fraction (LVEF) [23]. Intraoperative factors included duration of anesthesia and surgical intervention, as well as the type of procedure, categorized as isolated valve surgery, isolated on- (OPCAB) and off-pump coronary artery bypass grafting (CABG), combined CABG and other procedures, aortic surgery, ventricular assist device (VAD) implantation, and heart transplantation. The application of vasopressors or inotropic agents was quantified using the vasoactive inotropic score (VIS) [24]. Transfusion of red blood cells, platelets, or fresh frozen plasma was recorded, as well as the general use and overall duration of aortic cross clamping. Postoperative variables comprised length of stay in the intensive care unit (ICU) and the total hospital length of stay (LOS). Postoperative complications – including any infection (POI), acute kidney injury (AKI) according to the Kidney Disease: Improving Global Outcomes (KDIGO) criteria and a failure to extubate after 8 h of mechanical ventilation – were additionally assessed [25, 26]. 

Subgroup patients provided detailed data on frailty and postoperative delirium. Preoperative assessment included physical and cognitive frailty scoring, using an adaptation of the Fried Frailty Score and the Mini-Cog©, which allowed categorization of patients as robust, pre-frail, or frail, and identification of lower or higher likelihood of clinically significant cognitive impairment. Postoperative delirium is also recorded in subgroup patients. The frailty score includes gait-speed, assessed via timed-up-and-go-test, handgrip strength measured with a dynamometer, evaluation of unintentional weight loss, subjective exhaustion, and physical activity. The Mini-Cog©-Score consists of a three-word-recall task and a clock-drawing-test [27–29]. 

### Statistical analysis

Using R version 4.4.1, the dataset was filtered to include only patients who underwent cardiac surgical procedures [30–32]. All variables were categorized as previously described. The primary endpoints were the Physical Component Summary (PCS) and Mental Component Summary (MCS), calculated according to the standard scoring protocol as described in the user manual [10]. Univariate analyses were performed using simple linear regression to evaluate the association between each variable and the respective endpoints. For each model, the unstandardized regression coefficient (B) and corresponding p-value were calculated to assess between-group differences in outcomes. Separate univariate analyses were performed for both PCS and MCS prior to conducting multivariable regression analyses to identify variables independently associated with health-related quality of life (HR-QoL). To identify factors associated with postoperative HR-QoL rather than to construct an outcome-optimizing predictive model, all investigated variables were included in the multivariate analysis independent of their significance in univariate testing. A two-sided significance level of α = 0.05 was applied. Given the exploratory nature of this study, no adjustment for multiple testing was performed. Key findings were subsequently visualized graphically to illustrate between-group differences and the overall influence of relevant predictors on the endpoints.

To assess the robustness of the multivariable regression results, internal validation was performed using bootstrap resampling. A total of 5,000 bootstrap samples were drawn with replacement from the original dataset, and the multivariable linear regression model was refitted in each iteration. Bootstrap-based standard errors and bias-corrected and accelerated (BCa) 95% confidence intervals were calculated for variables included in the final models. This approach accounts for sampling variability and potential deviations from normality of the estimator distribution.

## Results

### Baseline characteristics

Mean age of the cohort was 67.71 years, 28.4 % were female. 1-year-mortality in all patients receiving cardiac surgery was 5.55 %. For all demographic and periprocedural data of the cohort and subgroup, see Table 1. In this cohort of cardiac surgery patients, the median EuroSCORE II – a risk score for perioperative mortality – was 1.60 %, reflecting overall low operative risk [33]. 

**Table 1:** Demographic and periprocedural baseline data of the analyzed cohort and additional data on physical and cognitive frailty in the subgroup (AKI: acute kidney injury; BMI: body mass index; Dur.: duration; FFP: fresh frozen plasma; ICU: intensive care unit; LDL: low density lipoprotein; Lp(a): lipoprotein(a); LVEF: left ventricular ejection fraction; NYHA: New York Heart Association; PAD: peripheral arterial disease; POH: postoperative hours; Postop.: postoperative; Preop.: preoperative; RBC: red blood cell; SD: standard deviation; VIS: vasoactive inotropic score)

Overall			Overall		
*n*	812		*n*	812	
Sex: Female (%)	231	(28.4)	Dur. anesthesia (minutes, mean (SD))	332.85	(70.27)
Age (mean (SD))	67.71	(9.62)	Dur. intervention (minutes, mean (SD))	211.51	(62.23)
Age-categories (%)			Type of surgery (%)		
18–64 years	285	(35.1)	Isolated valve	377	(46.4)
65–74 years	326	(40.1)	Isolated Off-Pump CABG	197	(24.3)
≥ 75 years	201	(24.6)	Isolated OPCAB	9	(1.1)
Missing	0	(0.0)	Aortic procedures	114	(14.0)
EuroSCORE II (%, median (IQR))	1.60	(2.42)	CABG combinations	101	(12.4)
Preop. hemoglobin (mg/dl, mean (SD))	14.09	(1.73)	VAD	4	(0.5)
Preop. anemia (%)			Others	10	(1.2)
None	681	(84.4)	Missing	0	(0.0)
Mild	94	(11.6)	Aortic Cross-Clamp (%)	583	(72.2)
Moderate or severe	32	(4.0)	≤ 60 min	104	(17.8)
Missing	5	(0.01)	61–120 min	368	(63.1)
Preop. LDL (mean (SD))			> 120 min	111	(19.0)
< 55 mg/dl	517	(64.9)	VIS (median (IQR); %)	9.1	(10.4)
55–69 mg/dl	97	(12.2)	VIS < 5	219	(27.0)
≥ 70 mg/dl	182	(22.9)	VIS 5–15	386	(47.5)
Missing	16	(0.02)	VIS > 15	206	(25.4)
Preop. Lp(a) ≥ 50 mg/dl (%)	271	(38.9)	Missing	1	(0.001)
BMI (mean (SD))	27.39	(5.20)	RBC transfusion (patients (%))	144	(17.7)
BMI-categories (%)			Platelet transfusion (patients (%))	177	(21.8)
Underweight	11	(1.4)	FFP transfusion (patients (%))	71	(8.7)
Normal weight	236	(29.5)	Any transfusion (patients (%))		
Overweight	320	(40.0)	None	550	(67.7)
Obese	233	(29.1)	1–2 transfusions	156	(19.2)
Missing	12	(0.02)	≥ 3 transfusions	106	(13.1)
Hypertension (%)	663	(81.7)	Dur. postop. ICU-stay (days, mean (SD))	2.21	(4.54)
Diabetes mellitus (%)	173	(21.3)	Time to extubation (hours, median (IQR))	8.02	(6.03)
PAD (%)	46	(5.7)	Failure to Extubate after 8 POH (%)	403	(50.3)
NYHA-classification (%)			Dur. in-hospital stay (days, mean (SD))	13.75	(9.36)
I	84	(10.4)	Postop. infection (%)	30	(3.7)
II	264	(32.6)	Postop. AKI (%)	133	(17.4)
III	440	(54.3)	Postop. RRT (%)	12	(1.5)
IV	22	(2.7)	Frailty-program (%)	190	(23.4)
Missing	2	(0.003)			
Smoking status (%)			n	190	
No smoking	447	(55.3)	Frailty-Score (%)		
Active smoking	115	(14.2)	Robust	67	(35.3)
Former smoking	246	(30.4)	Pre-Frail	92	(48.4)
Missing	4	(0.005)	Frail	31	(16.3)
LVEF (mean (SD))	54.48	(9.50)	Missing	0	(0.0)
LVEF-categories (%)			Mini-Cog©-Score (%)		
≥ 50%	674	(83.0)	Lower likelihood	151	(79.5)
41–49%	61	(7.5)	Higher likelihood	14	(7.4)
≤ 40%	77	(9.5)	Missing	25	(13.2)
Missing	0	(0.0)	Postop. delirium (%)	12	(6.3)

### Outcome

**Fig. 2:**
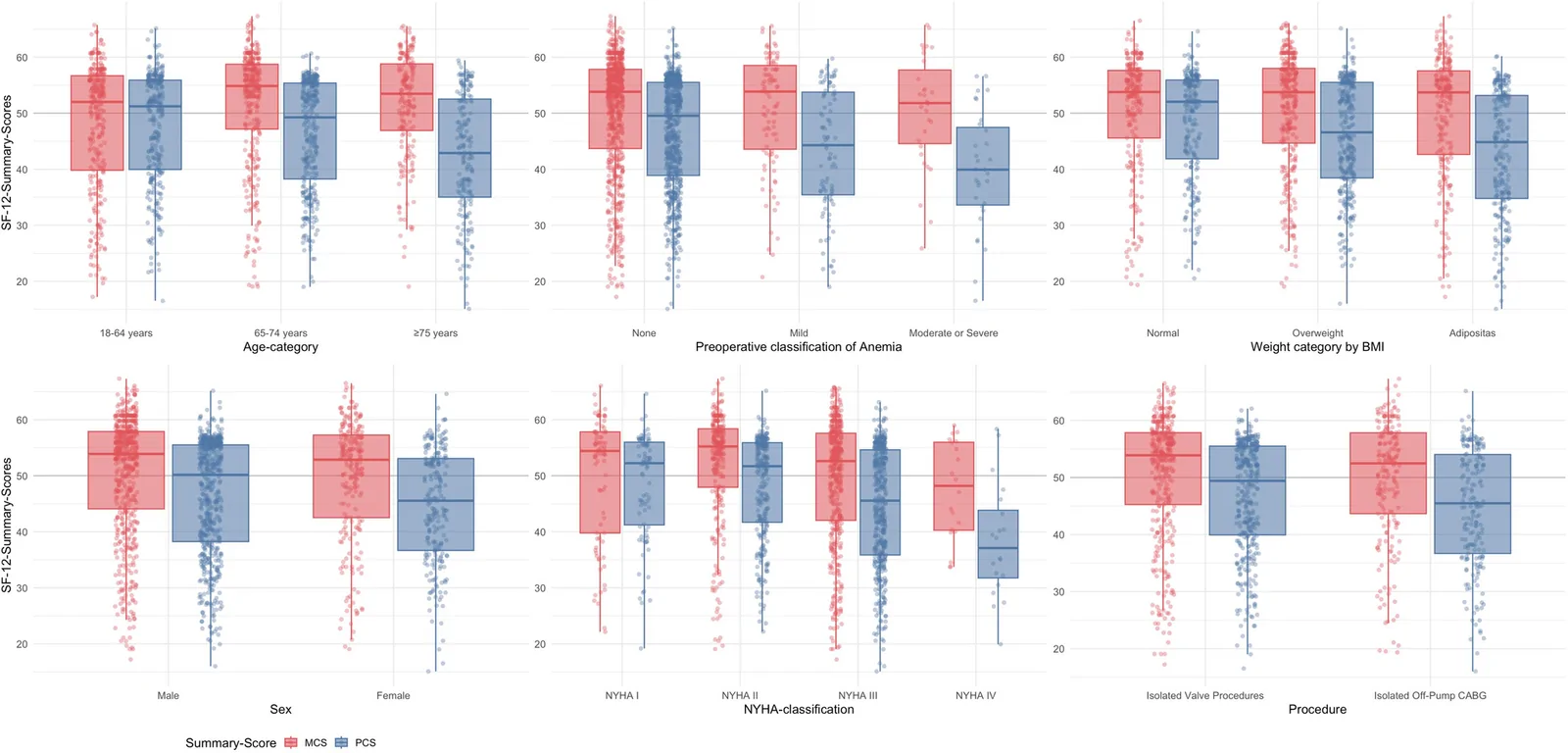
SF-12-scores by sex and age-category, grade of preoperative anemia, weight categorized by body-mass-index (BMI), symptom burden according to NYHA-classification and type of performed procedure (SF-12: Short-Form-12; MCS: mental component summary; PCS: physical component summary; BMI: body mass index; NYHA: New York Heart Association, CABG: coronary artery bypass graft)

Mean physical outcome (PCS) was 45.91 (SD 10.31) and mean mental outcome (MCS) was 50.12 (SD 10.71). Female sex [B = -1.8 (95% CI -3.3 – -0.2), *p* = 0.03] and increase in age [B = -5.0 (95% CI -6.8 – -3.1), *p* < 0.001] were associated with significantly lower PCS, while older age was associated with significantly higher MCS [B = 3.7 (95% CI 1.7–5.6), *p* < 0.001] (Fig. 2).

Patients preoperatively presenting with anemia [B = -6.8 (95% CI -10.5 – -3.2), *p* < 0.001], increased BMI [B = -5.1 (95% CI -6.9 – -3.2), *p* < 0.001], positive history of hypertension [B = -3.3 (95% CI -5.1 – -1.5), *p* < 0.001], diabetes mellitus [B = -2.9 (95% CI -4.6 – -1.2), *p* < 0.01], vascular comorbidities [B = -4.6 (95% CI -7.7 – -1.5), *p* < 0.01] and smoking [B = -2.2 (95% CI -3.8 – -0.6), *p* < 0.01] presented significantly lower physical outcome scores – smoking having an additional negative association with MCS [B = -2.1 (95% CI -3.7 – -0.4), *p* = 0.02]. Increase in NYHA-classification [B = -10.9 (95% CI -15.6 – -6.2), *p* < 0.001] and decrease in LVEF [B = -3.8 (95% CI -6.3 – -1.4), *p* < 0.01] were also associated with significantly lower PCS (Fig. 2).

Prolonged duration of anesthesia [B = -0.02 (95% CI -0.03 – -0.01), *p* < 0.01] and surgical intervention [B = -0.01 (95% CI -0.02 – -0.001), *p* = 0.03], as well as prolonged ICU-stay [B = -0.4 (95% CI -0.6 – -0.3), *p* < 0.001] and total length of stay [B = -0.2 (95% CI -0.3 – -0.1), *p* < 0.001], were all associated with significant lower physical outcome scores (PCS). Compared with isolated valve procedures, patients undergoing isolated Off-Pump CABG also exhibited lower physical outcome scores [B = -2.0 (95% CI -3.75 – -0.26), *p* = 0.02] (Fig. 2), with no relevant differences in mean age or preoperative risk evaluation (EuroSCORE II) but a higher prevalence of PAD in patients undergoing off-pump CABG (11.2% vs. 3.7%; *p* < 0.001). Patients undergoing isolated OPCAB, heart transplantation or VAD-implantation were excluded from this analysis due to insufficient sample size. The intraoperative use of an aortic cross-clamp was associated with higher PCS [B = 2.4 (95% CI 0.8–4.0), *p* = 0.003], while the overall duration of clamping showed no association with HR-QoL in outcome. The intraoperative transfusion of RBC, platelets or FFP was associated with significantly lower physical scores [B = -4.7 (95% CI -6.8 – -2.5), *p* < 0.001]. An increased application of vasoactive or inotropic agents was not associated with differences in physical or mental outcome.

**Fig. 3:**
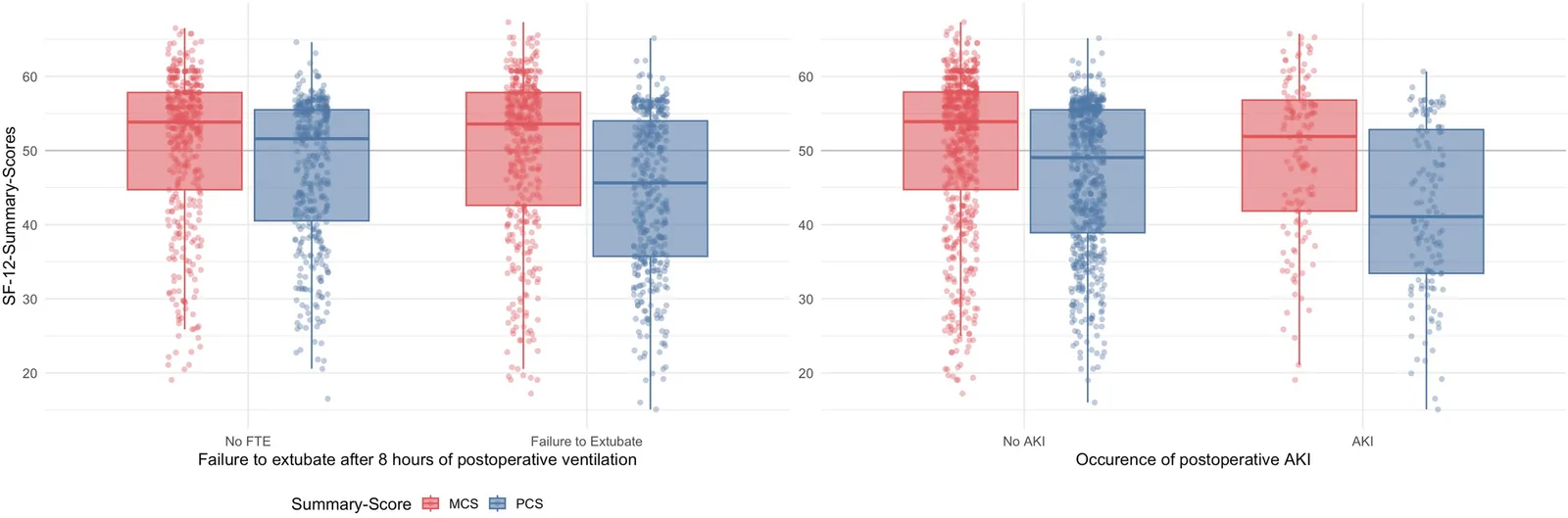
SF-12-scores by duration of postoperative ventilation and occurrence of postoperative acute kidney injury (AKI: acute kidney injury; FTE: failure to extubate; MCS: mental component summary; PCS: physical component summary; SF-12: Short-Form-12)

Patients undergoing any postoperative infection (POI) presented significantly lower PCS [B = -7.8 (95% CI -11.5 – -4.1), *p* < 0.001] and MCS [B = -4.2 (95% CI -8.1 – -0.3), *p* = 0.03]. A prolonged mechanical ventilation, defined as a failure to extubate after 8 postoperative hours was associated with a significantly lower PCS [B = -3.1 (95% CI -4.5 – -1.7 ), *p* < 0.0001], as was a postoperative acute kidney injury, defined according to the KDIGO-guidelines using serum creatinine and urine output [B = -4.5 (95% CI -6.4 – -2.6), *p* < 0.0001] (Figure 3).

**Fig. 4:**
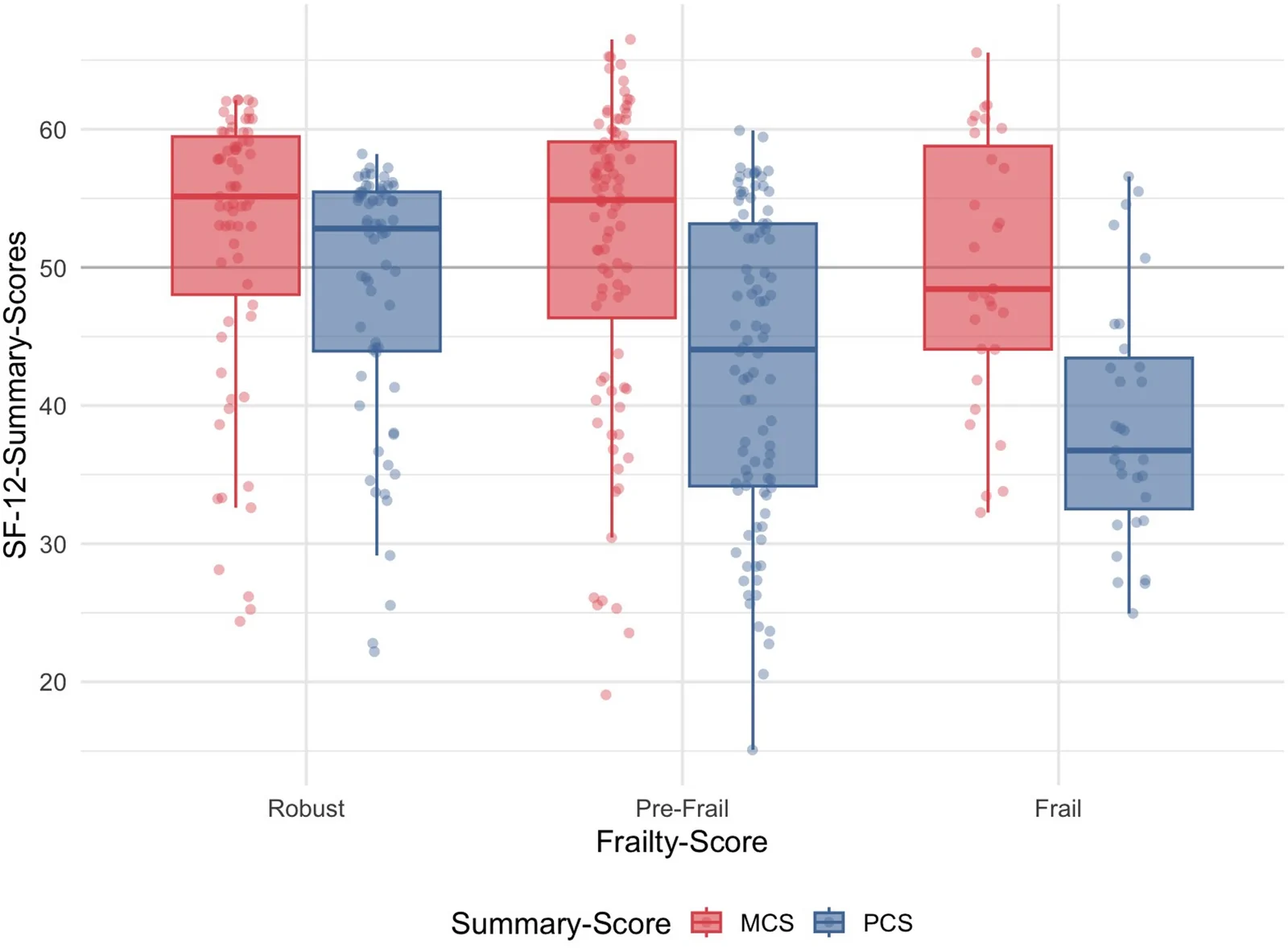
SF-12-scores by classification of physical frailty (SF-12: Short-Form-12; MCS: mental component summary; PCS: physical component summary)

Participation in the frailty- and delirium-pathway was associated with significantly lower PCS [B = -2.2 (95% CI -3.9 – -0.6), *p* < 0.01], as was preoperative classification as physically pre-frail [B = -5.6 (95% CI -8.8 – -2.4), *p* < 0.01] or frail [B = -9.6 (95% CI -14.0 – -5.2), *p* < 0.001] (Fig. 4 , Supplementary Table 1).

**Fig. 5:**
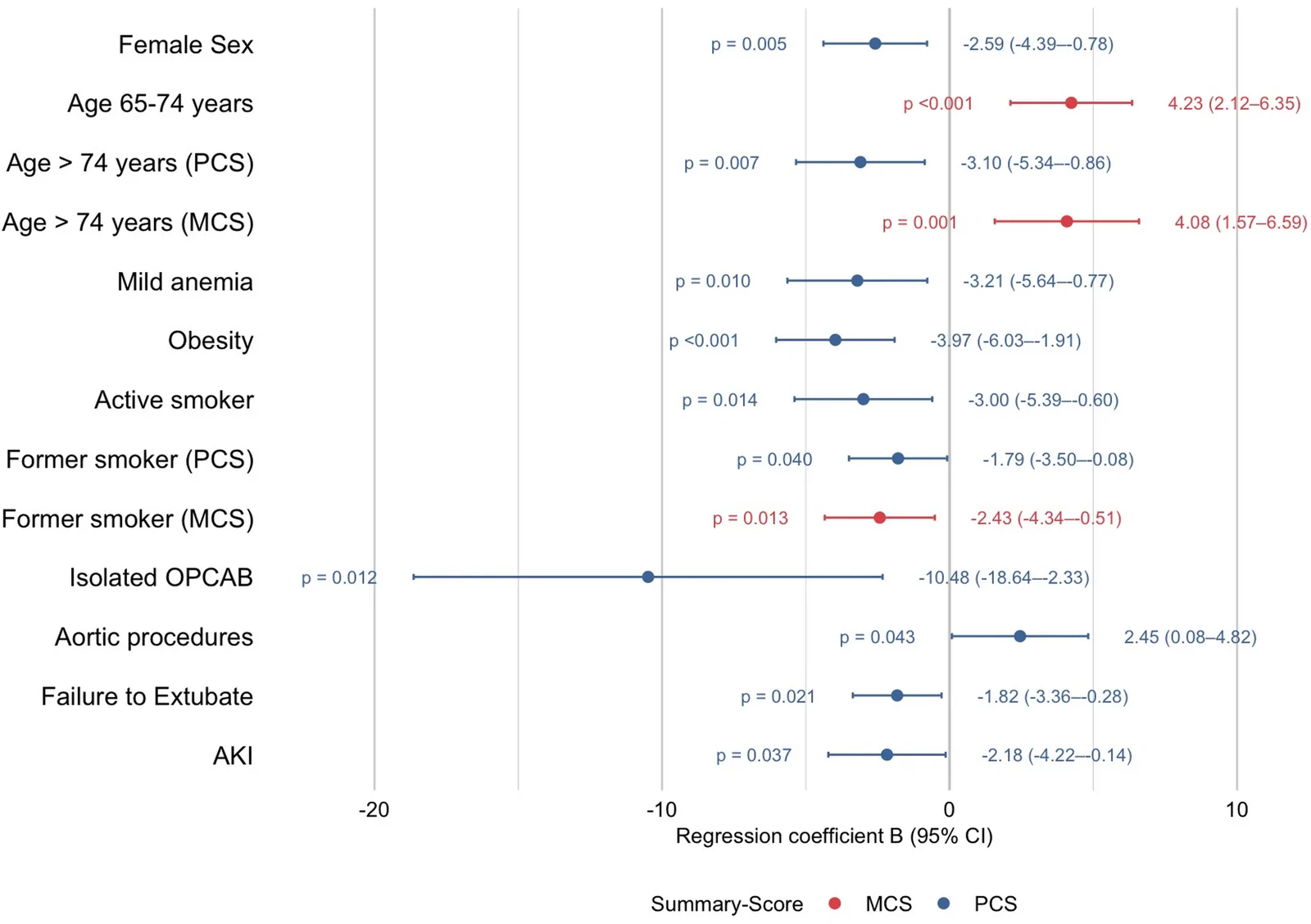
Factors independently associated with differences in postoperative HR-QoL as identified in multivariate analyses (AKI: acute kidney injury; CI: confidence interval; MCS: mental component summary; PCS: physical component summary; PAD: peripheral arterial disease; NYHA: New York Heart Association; CABG: coronary artery bypass graft)

A multivariate analysis identified female sex, age < 74 years, a preoperative anemia, obesity, a history of smoking were identified as independent preoperative predictors of lower physical outcome scores, as assessed by the SF-12-questionnaire. Concerning intra- and postoperative characteristics, performance of aortic procedures, failure to extubate after 8 h of postoperative ventilation and postoperative acute kidney injury (AKI) were associated with independently poorer PCS. A history of smoking independently predicted lower cognitive outcome scores, while increased age was independently associated with significantly higher MCS (Fig. 5, Supplementary Table 2).

### Subgroup analysis: Frail patients

**Fig. 6:**
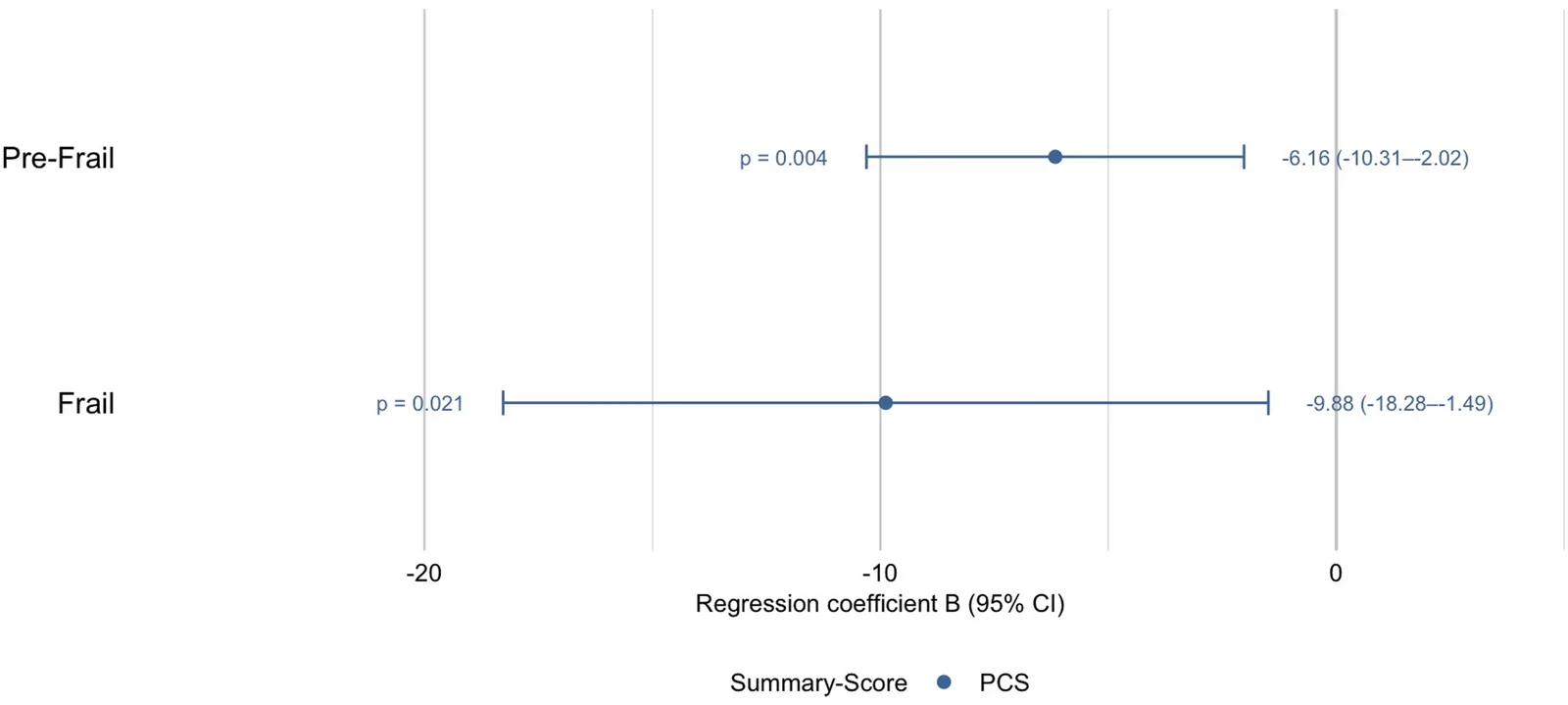
Factors independently associated with differences in postoperative HR-QoL as identified in multivariate analyses in the subgroup (HR-QoL: health-related quality-of-life; SF-12: Short-Form-12; MCS: mental component summary; PCS: physical component summary)

The distribution of frailty status showed no significant difference between off-pump bypasses and isolated valve procedures. A multivariate subgroup-analysis identified preoperative physical pre-frailty and frailty as independent predictors of lower physical outcome scores (Fig. 6 , Supplementary Table 3). Cognitive frailty was not associated with significant changes in PCS or MCS .

### Internal validation

Internal validation using bootstrap resampling confirmed the robustness of several associations identified in the primary analysis. Female sex, advanced age, obesity, history of smoking, preoperative anemia, failure to extubate within 8 h postoperatively, and postoperative acute kidney injury showed consistent associations with lower physical HR-QoL scores. Physical pre-frailty and frailty were also consistently associated with lower physical outcome scores. For cognitive HR-QoL (MCS), associations with advanced age and smoking history remained consistent across resampling (Supplementary Table 4).

## Discussion

The results of this prospective observational study demonstrate several important findings regarding prediction of health-related quality-of-life in patients undergoing cardiac surgery.

Multivariate analyses revealed an independent association of female sex, an increase in age and weight, preoperative anemia, history of smoking, performance of aortic procedures, as well as postoperative AKI or failure to extubate after 8 hours of postoperative ventilation with poorer physical outcome scores. Regarding mental outcome, older age was associated with higher outcome scores, while a history of smoking was linked to an impairment of mental scoring. A subgroup analysis identified physical frailty and pre-frailty as independent predictors of impaired physical outcome.

According to user manual, the SF-12-scores are norm based and standardized to the general population with a mean of 50 and a standard deviation of 10, lower scores reflecting poorer outcome [10, 12]. In this analysis, mean physical outcome was reduced by approximately half a standard deviation (45.91), whereas mean mental outcome did not differ from that of the general population (50.12). A comparable pattern emerged in the relationship between age and outcomes: older age was associated with impaired physical health, whereas mental health tended to improve with age. These findings may indicate that physical health is generally more susceptible to influencing factors, such as surgery, than mental health.

Older patients presented significantly lower scores in physical outcome but showed higher scores in cognitive outcome. This may be due to a ceiling effect, attributable to a difference in preoperative scores, and varying expectations of outcome, as well as psychosocial challenges [34, 35]. A higher preoperative HR-QoL and greater expectations regarding postoperative quality of life in younger patients leave less room for measurable improvement, whereas older patients may be more easily satisfied with postoperative outcomes, potentially resulting in differing postoperative scores. Younger patients often report higher expectations regarding full recovery and experience greater preoperative psychological distress, which has been associated with worse short-term postoperative outcomes [36, 37]. However, these findings are not consistently supported by existing literature, which more frequently reports either no significant association between age and mental HR-QoL or less favorable trajectories in older patients [34, 38]. 

Preoperative anemia and frailty, both independently associated with worse outcomes, represent potentially modifiable risk factors whose optimization could reduce complications and improve postoperative results. Patient Blood Management (PBM), which aims to optimize red blood cell mass, minimize blood loss, and enhance a patient’s tolerance to anemia, constitutes an important strategy for the structural management of perioperative anemia and has been shown to have clinically relevant effects on outcomes [16, 17]. In our analysis, preoperative anemia was confirmed as a significant predictor of postoperative outcome, underscoring the ongoing need to implement and refine PBM strategies. Preoperative frailty deems a similar approach of perioperative management: an enhanced preoperative assessment of cognition, function and frailty, perioperative support including family, and postoperative care focusing on delirium-prevention and functional recovery are recommended to minimize complications and improve outcome [39, 40]. The observed association between physical frailty and impaired physical outcomes supports evidence linking frailty to adverse events, including increased mortality, prolonged hospital stays, and higher risk of delirium [41–43]. By focusing on preoperative optimization and extended risk stratification to reduce perioperative complications and overall length of stay, physicians may help mitigate the economic burden for patients undergoing complex procedures, particularly those with extensive comorbidities or exhibiting physical or mental vulnerability [44, 45]. 

Postoperative complications, including a failure to extubate after 8 hours of postoperative ventilation and a postoperative acute kidney injury (AKI), were independently associated with lower physical outcome scores during 1-year-follow-up. Complications warrant further medical intervention, enhanced treatment and have previously been identified as predictors of mortality and length of hospital-stay [46, 47]. In this cohort, 5 patients (0.6 %) underwent a postoperative mechanical ventilation of ≥ 48 hours, warranting a classification by ability to extubate after 8 hours, as previously described by Yende and Wunderink, which corresponds with this cohort’s median time to extubation [26]. Previous studies also assessed the association of renal replacement therapy (RRT) with HR-QoL [48]. In this cohort, the incidence of postoperative RRT was 1.5 % (12 of 806 patients), warranting no reliable analysis.

Overall, the results of this analysis are in line with previously identified predictors of HR-QoL. A systematic review, published by Sanders et al. in 2022, identified 103 independent predictors of HR-QoL after cardiac surgery, including BMI, smoking and length of stay in the hospital and on the ICU, while emphasizing a lack of consistency across studies [13]. A follow-up study on 272 patients, published by Perrotti et al. in 2019, demonstrated the association between dyspnea, older age and impaired physical outcome [49]. Regarding this analysis’ subgroup, an observational study on 133 patients, published by Nakano et al. in 2020, revealed an association of frailty with functional decline after cardiac surgery [22]. All findings suggest a distinct phenotype at risk for impaired outcome after surgery, which could benefit from extensive patient-centered care and perioperative management.

This study presents both strengths and weaknesses. By analyzing a sample size comparable to similar studies, it contributes further evidence on the prediction of HR-QoL after cardiac surgery and adds to the consistency in existing literature, while emphasizing the vulnerability of frail patients undergoing high-risk cardiac surgery. These findings may have broader implications for cardiac surgical patients and underscore the relevance of partially modifiable perioperative factors. Several limitations should be acknowledged. The single-center design and the lack of preoperative HR-QoL assessment limit the interpretation of postoperative findings. Baseline assessment would have allowed for the evaluation of absolute changes in quality of life in addition to relative between-group differences. Furthermore, it could have provided insight into the predictive value of baseline HR-QoL and helped to identify potential statistical moderators. Preoperative HR-QoL may have acted as a potential confounder or effect modifier, potentially influencing the observed magnitude of postoperative differences. Without baseline assessment, it cannot be fully excluded that part of the differences in postoperative HR-QoL may reflect pre-existing differences in patient status rather than solely postoperative effects or recovery trajectories. During follow-up, 187 patients (16.5 %) did not return the questionnaire, and 62 (5.5 %) returned an incomplete questionnaire. In addition, 75 patients (6.6 %) were deceased at follow-up or excluded for other reasons, resulting in a total dropout of 324 patients (28.5 %), which may introduce substantial bias. Selection bias—arising from the inclusion of only patients who were able and willing to participate—and response bias—since immediate outcomes, mortality, or disability may have influenced questionnaire return—may have affected the results. Given the exploratory nature and the absence of adjustment for multiple testing, the reported p-values should be interpreted with caution and primarily considered hypothesis-generating rather than confirmatory. Finally, no formal a priori sample size calculation was performed, as the study was designed as an exploratory prospective observational cohort including all consecutive eligible patients during the study period. The primary aim was to assess associations between perioperative variables and continuous postoperative HR-QoL using regression-based analyses rather than hypothesis testing between predefined groups. Accordingly, formal sample size estimation based on group comparisons was not considered appropriate for the present study design.

To evaluate the stability of the identified associations, bootstrap-based internal validation was performed for selected variables that demonstrated statistically significant and consistent effects in the primary multivariable analyses. The results largely confirmed the robustness of key associations, including female sex, advanced age, obesity, smoking status, preoperative anemia, failure to extubate, postoperative acute kidney injury, and physical frailty in relation to lower postoperative physical HR-QoL. For cognitive HR-QoL, associations with advanced age and smoking status were consistently observed across resampling. This procedure represents an internal validation approach only and does not constitute external validation. While bootstrap resampling assesses the stability of the observed associations within the study sample, it does not account for between-population heterogeneity or differences in clinical settings. Therefore, the findings should be interpreted as internally validated but not yet generalizable. External validation in independent, preferably multicenter cohorts is required to confirm reproducibility and generalizability.

Future research should incorporate preoperative assessment of HR-QoL to evaluate absolute effects on QoL and should be designed as multicenter studies to include a broader patient spectrum, increase sample size, and enhance generalizability of the findings. Furthermore, identification of high-risk patients is possible, but clinical translation remains crucial for improving care and optimizing outcome; subjective, objective and economically.

## Conclusion

In patients undergoing cardiac surgery at a high-volume German heart center, female sex, advanced age, higher body weight, preoperative anemia, smoking history, prolonged postoperative mechanical ventilation > 8 hours, and acute kidney injury were independently associated with reduced physical HR-QoL at 1-year follow-up. Increased age was positively associated with cognitive outcome, whereas smoking was linked to impaired cognitive scores. Physical frailty emerged as an independent predictor of poorer physical HR-QoL. These findings suggest a high-risk phenotype and highlight the need for improved perioperative risk stratification and targeted management of vulnerable patients.

## Supplementary Information

Supplementary Material 1.

## Data Availability

The datasets used and/or analyzed during the current study are available from the corresponding author on reasonable request.

## Abbreviations

AKI: Acute Kidney Injury
ANQUR: Anesthesiology-QUality-Registry
BMI: Body Mass Index
CABG: Coronary Artery Bypass Graft
CI: Confidence Interval
ERAS: Enhanced Recovery after Surgery
FTE: Failure to Extubate
HR-QoL: Health-Related Quality-of-Life
ICU: Intensive Care Unit
KDIGO: Kidney Disease: Improving Global Outcomes
LDL: Low Density Lipoprotein
LOS: Length of Stay
Lp(a): Lipoprotein (a)
LVEF: Left Ventricular Ejection Fraction
MCS: Mental Component Summary
NYHA: New York Heart Association
OPCAB: Off-Pump Coronary Artery Bypass
PAD: Peripheral Arterial Disease
PBM: Patient Blood Management
PCS: Physical Component Summary
POH: Postoperative Hours
POI: Postoperative Infection
PRO: Patient-Reported Outcome
SD: Standard Deviation
SF-12: Short-Form 12
VAD: Ventricular Assist Device
VIS: Vasoactive Inotropic Score
WHO: World Health Organization
