# Supplementary material for: Predictors of health-related quality-of-life after cardiac surgery: findings from the ANesthesiology-QUality-Registry (ANQUR) and frailty-management
Source: BMC Anesthesiol. 2026 May 25;26:332. doi: 10.1186/s12871-026-03931-8 (PMC13202746; doi:10.1186/s12871-026-03931-8)
Supplement: Supplementary file 1 — Supplementary Material 1. [file 12871_2026_3931_MOESM1_ESM.docx]

**Supplements**

|  | **PCS** | | **MCS** | |
| --- | --- | --- | --- | --- |
| **Variable** | **Estimate (95 % CI)** | **p-value** | **Estimate (95 % CI)** | **p-value** |
| Female sex | -1.749 (-3.322 – -0.177) | **0.029** | -0.87 (-2.507 – 0.767) | 0.297 |
| Age | -0.182 (-0.255 – -0.109) | **< 0.001** | 0.134 (0.058 – 0.211) | **0.001** |
| Age 65-74 y. | -1.259 (-2.861 – 0.343) | 0.123 | 3.538 (1.867 – 5.209) | **< 0.001** |
| Age ≥ 75 y. | -4.963 (-6.821 – -3.105) | **< 0.001** | 3.657 (1.719 – 5.595) | **< 0.001** |
| Preop. Hb | 1.058 (0.652 – 1.464) | **< 0.001** | -0.077 (-0.505 – 0.352) | 0.725 |
| Mild anemia | -3.071 (-5.272 – -0.871) | **0.006** | 0.622 (-1.691 – 2.935) | 0.598 |
| Mod./sev. an. | -6.831 (-10.448 – -3.214) | **< 0.001** | 0.993 (-2.809 – 4.796) | 0.608 |
| LDL 55-69 mg/dl | 0.167 (-2.064 – 2.398) | 0.883 | -0.391 (-2.721 – 1.94) | 0.742 |
| LDL ≥ 70 mg/dl | -0.919 (-2.657 – 0.819) | 0.300 | 0.919 (-0.897 – 2.734) | 0.321 |
| Lp(a) ≥ 50 mg/dl | 0.729 (-0.845 – 2.303) | 0.364 | 0.732 (-0.909 – 2.373) | 0.381 |
| Underweight | -1.442 (-7.595 – 4.71) | 0.645 | -1.971 (-8.461 – 4.519) | 0.551 |
| Overweight | -2.217 (-3.929 – -0.506) | **0.011** | 0.384 (-1.421 – 2.189) | 0.676 |
| Obese | -5.063 (-6.905 – -3.221) | **< 0.001** | -0.481 (-2.424 – 1.462) | 0.627 |
| Hypertension | -3.27 (-5.094 – -1.445) | **< 0.001** | -0.305 (-2.215 – 1.605) | 0.754 |
| Diabetes | -2.904 (-4.63 – -1.178) | **0.001** | -0.283 (-2.088 – 1.522) | 0.759 |
| PAD | -4.594 (-7.655 – -1.533) | **0.003** | 0.763 (-2.434 – 3.961) | 0.639 |
| Active smoking | -1.833 (-3.938 – 0.272) | 0.088 | -1.975 (-4.166 – 0.215) | 0.077 |
| Former smoking | -2.183 (-3.782 – -0.585) | **0.007** | -2.062 (-3.725 – -0.399) | **0.015** |
| NYHA II | -0.382 (-2.861 – 2.098) | 0.763 | 1.991 (-0.628 – 4.611) | 0.136 |
| NYHA III | -3.965 (-6.322 – -1.608) | **0.001** | -0.774 (-3.264 – 1.715) | 0.542 |
| NYHA IV | -10.885 (-15.626 – -6.145) | **< 0.001** | -3.706 (-8.713 – 1.302) | 0.147 |
| LVEF | 0.149 (0.075 – 0.223) | **< 0.001** | 0.018 (-0.06 – 0.096) | 0.652 |
| LVEF 41 - 49 % | -2.17 (-4.864 – 0.524) | 0.114 | 0.588 (-2.229 – 3.405) | 0.682 |
| LVEF ≤ 40 % | -3.832 (-6.256 – -1.408) | **0.002** | -0.688 (-3.222 – 1.847) | 0.595 |
| Dur. anesth. | -0.017 (-0.027 – -0.007) | **0.001** | 0 (-0.01 – 0.011) | 0.959 |
| Dur. interv. | -0.013 (-0.024 – -0.001) | **0.027** | 0.002 (-0.009 – 0.014) | 0.692 |
| Isolated Off-Pump CABG | -2.006 (-3.75 – -0.261) | **0.024** | -0.894 (-2.738 – 0.951) | 0.342 |
| CABG combinations | -1.635 (-3.874 – 0.604) | 0.152 | 0.31 (-2.04 – 2.661) | 0.796 |
| Aortic procedures | 0.901 (-1.234 – 3.037) | 0.408 | 0.048 (-2.194 – 2.291) | 0.966 |
| Dur. ICU-stay | -0.423 (-0.577 – -0.269) | **< 0.001** | -0.084 (-0.247 – 0.079) | 0.313 |
| Dur. hosp.-stay | -0.203 (-0.278 – -0.128) | **< 0.001** | -0.047 (-0.125 – 0.032) | 0.248 |
| POI | -7.782 (-11.516 – -4.048) | **< 0.001** | -4.231 (-8.138 – -0.323) | **0.034** |
| Frailty-program | -2.226 (-3.9 – -0.552) | **0.009** | 1.626 (-0.116 – 3.368) | 0.067 |
| Pre-Frail | -5.576 (-8.808 – -2.345) | **0.001** | -0.758 (-4.092 – 2.577) | 0.655 |
| Physical frailty | -9.586 (-13.957 – -5.216) | **< 0.001** | -2.245 (-6.755 – 2.265) | 0.327 |
| Mental frailty | -5.182 (-11.017 – 0.652) | 0.081 | -4.723 (-10.499 – 1.052) | 0.108 |
| POD | 1.174 (-5.145 – 7.494) | 0.714 | 0.349 (-5.843 – 6.54) | 0.912 |
| AXC | 2.528 (0.949 – 4.106) | **0.002** | 1.553 (-0.093 – 3.199) | 0.064 |
| 61-120 min. AXC | 0.389 (-1.832 – 2.609) | 0.731 | 0.559 (-1.730 – 2.847) | 0.632 |
| > 120 min. AXC | -1.469 (-4.202 – 1.264) | 0.292 | 1.305 (-1.512 – 4.122) | 0.363 |
| 1-2 transfusions | - 2.075 (-3.893 – - 0.258) | **0.025** | 2.269 (-0.377 – 4.915) | 0.927 |
| ≥ 3 transfusions | -4.674 (-6.799 – -2.548) | **< 0.0001** | 0.411 (-1.819 – 2.640) | 0.718 |
| VIS 5-15 | -0.604 (-2.318 – 1.111) | 0.490 | 0.510 (-1.272 – 2.292) | 0.574 |
| VIS > 15 | -1.360 (-3.327 – 0.607) | 0.175 | 1.017 (-1.027 – 3.062) | 0.329 |
| Failure to Extubate | -3.122 (-4.537 – -1.707) | **< 0.0001** | -0.825 (-2.308 – 0.658) | 0.275 |
| AKI | -4.544 (-6.459 – -2.629) | **< 0.0001** | 0.872 (-1.145 – 2.889) | 0.396 |
|  |  |  |  |  |

Supplementary Table 1: Univariate analyses (CABG: coronary artery bypass graft; CI: confidence interval; Dur.: duration; Hb: hemoglobin; LDL: low density lipoprotein; Lp(a): lipoprotein(a); LVEF: left ventricular ejection fraction; MCS: mental component summary; Mod./sev. an.: moderate/severe anemia; NYHA: New York Heart Association; PAD: peripheral arterial disease; PCS: physical component summary; POI: postoperative infection; POD: postoperative delirium; Preop.: preoperative; y.: years)

|  | **PCS** | | **MCS** | |
| --- | --- | --- | --- | --- |
| **Variable** | **Estimate (95 % CI)** | **p-value** | **Estimate (95 % CI)** | **p-value** |
| Female sex | -2.585 (-4.388 – -0.783) | **0.005** | -1.018 (-3.039 – 1.004) | 0.323 |
| Age 65-74 y. | -0.222 (-2.109 – 1.665) | 0.817 | 4.234 (2.117 – 6.35) | **<0.001** |
| Age ≥ 75 y. | -3.1 (-5.338 – -0.863) | **0.007** | 4.081 (1.572 – 6.59) | **0.001** |
| Mild anemia | -3.207 (-5.64 – -0.775) | **0.01** | -1.32 (-4.048 – 1.408) | 0.342 |
| Mod./sev. an. | -2.477 (-7.037 – 2.082) | 0.286 | 0.503 (-4.611 – 5.616) | 0.847 |
| LDL 55-69 mg/dl | 1.937 (-0.426 – 4.3) | 0.108 | 0.339 (-2.311 – 2.989) | 0.801 |
| LDL ≥ 70 mg/dl | 0.545 (-1.302 – 2.391) | 0.563 | 1.08 (-0.991 – 3.151) | 0.306 |
| Lp(a) ≥ 50 mg/dl | 0.151 (-1.394 – 1.696) | 0.848 | 0.308 (-1.424 – 2.041) | 0.727 |
| Underweight | 0.867 (-5.714 – 7.449) | 0.796 | -3.784 (-11.165 – 3.597) | 0.314 |
| Overweight | -1.439 (-3.34 – 0.461) | 0.137 | 1.235 (-0.897 – 3.366) | 0.256 |
| Obese | -3.971 (-6.031 – -1.912) | **<0.001** | 0.852 (-1.458 – 3.162) | 0.469 |
| Hypertension | -1.606 (-3.652 – 0.439) | 0.124 | -0.669 (-2.962 – 1.625) | 0.567 |
| Diabetes | -1.566 (-3.51 – 0.377) | 0.114 | 0.169 (-2.01 – 2.349) | 0.879 |
| PAD | -3.22 (-6.459 – 0.018) | 0.051 | 1.521 (-2.112 – 5.153) | 0.411 |
| Active smoking | -2.998 (-5.394 – -0.603) | **0.014** | -2.141 (-4.828 – 0.545) | 0.118 |
| Former smoking | -1.791 (-3.498 – -0.083) | **0.04** | -2.426 (-4.341 – -0.511) | **0.013** |
| NYHA II | -0.006 (-2.716 – 2.704) | 0.996 | 0.079 (-2.961 – 3.118) | 0.959 |
| NYHA III | -2.004 (-4.641 – 0.633) | 0.136 | -2.124 (-5.082 – 0.833) | 0.159 |
| NYHA IV | -4.716 (-10.328 – 0.896) | 0.099 | -3.964 (-10.258 – 2.331) | 0.217 |
| LVEF 41 - 49 % | -0.474 (-3.286 – 2.338) | 0.741 | 0.843 (-2.31 – 3.997) | 0.600 |
| LVEF ≤ 40 % | 0.146 (-2.615 – 2.908) | 0.917 | 1.665 (-1.432 – 4.762) | 0.291 |
| Isolated Off-Pump CABG | -0.144 (-5.219 – 4.932) | 0.956 | -8.032 (-17.178 – 1.115) | 0.085 |
| CABG combinations | -0.616 (-3.138 – 1.907) | 0.632 | -0.895 (-3.724 – 1.934) | 0.535 |
| Aortic procedures | -0.616 (-3.138 – 1.907) | **0.043** | 0.529 (-2.129 – 3.187) | 0.696 |
| POI | -2.52 (-6.654 – 1.613) | 0.232 | -2.676 (-7.312 – 1.96) | 0.257 |
| AXC | 1.352 (-3.362 – 6.066) | 0.574 | 0.107 (-5.18 – 5.394) | 0.968 |
| 1-2 transfusions | -1.113 (-3.146 – 0.921) | 0.283 | 1.494 (-0.787 – 3.775) | 0.199 |
| ≥ 3 transfusions | -1.576 (-4.196 – 1.044) | 0.238 | -0.47 (-3.409 – 2.468) | 0.753 |
| VIS 5-15 | -0.78 (-2.717 – 1.158) | 0.43 | 0.58 (-1.593 – 2.753) | 0.600 |
| VIS > 15 | 0.369 (-1.995 – 2.732) | 0.759 | 2.357 (-0.294 – 5.008) | 0.081 |
| Failure to Extubate | -1.822 (-3.365 – -0.28) | **0.021** | -1.253 (-2.983 – 0.477) | 0.155 |
| AKI | -2.176 (-4.216 – -0.137) | **0.037** | -0.626 (-2.914 – 1.661) | 0.591 |
| Frailty-program | -0.213 (-2.221 – 1.796) | 0.835 | 0.192 (-2.06 – 2.445) | 0.867 |
|  | R^2^ / R^2^ adjusted 0.219 / 0.172 | | R^2^ / R^2^ adjusted 0.094 / 0.041 | |
|  |  |  |  |  |

Supplementary Table 2: Multivariate analysis of the cohort (CABG: coronary artery bypass graft; CI: confidence interval; LDL: low density lipoprotein; Lp(a): lipoprotein(a); LVEF: left ventricular ejection fraction; MCS: mental component summary; Mod./sev. an.: moderate/severe anemia; NYHA: New York Heart Association; PAD: peripheral arterial disease; PCS: physical component summary; POI: postoperative infection; y.: years)

|  | **PCS** | | **MCS** | |
| --- | --- | --- | --- | --- |
| **Variable** | **Estimate (95 % CI)** | **p-value** | **Estimate (95 % CI)** | **p-value** |
| Female sex | -1.356 (-5.357 – 2.644) | 0.503 | -1.549 (-5.939 – 2.841) | 0.486 |
| Age ≥ 75 y. | -1.532 (-5.291 – 2.227) | 0.421 | 0.837 (-3.288 – 4.963) | 0.688 |
| Mild anemia | -6.269 (-11.438 – -1.1) | 0.018 | -4.147 (-9.819 – 1.526) | 0.15 |
| Mod./sev. an. | -2.93 (-11.149 – 5.289) | 0.482 | 0.205 (-8.815 – 9.224) | 0.964 |
| LDL 55-69 mg/dl | 4.494 (-1.73 – 10.719) | 0.155 | 1.244 (-5.586 – 8.075) | 0.719 |
| LDL ≥ 70 mg/dl | 1.627 (-2.315 – 5.569) | 0.415 | 1.04 (-3.285 – 5.366) | 0.635 |
| Lp(a) ≥ 50 mg/dl | 1.229 (-2.315 – 4.772) | 0.494 | -0.908 (-4.796 – 2.981) | 0.645 |
| Underweight | -1.646 (-10.969 – 7.677) | 0.727 | -2.562 (-12.793 – 7.669) | 0.621 |
| Overweight | 0.874 (-3.791 – 5.539) | 0.711 | 1.521 (-3.598 – 6.641) | 0.557 |
| Obese | -6.464 (-11.924 – -1.004) | **0.021** | -2.176 (-8.168 – 3.816) | 0.473 |
| Hypertension | 0.827 (-4.895 – 6.549) | 0.775 | -0.053 (-6.332 – 6.226) | 0.987 |
| Diabetes | 2.093 (-2.881 – 7.067) | 0.406 | 2.525 (-2.934 – 7.984) | 0.361 |
| PAD | -4.031 (-10.184 – 2.122) | 0.197 | -3.04 (-9.792 – 3.712) | 0.374 |
| Active smoking | -1.139 (-8.2 – 5.922) | 0.75 | -1.223 (-8.972 – 6.526) | 0.755 |
| Former smoking | -2.055 (-6.138 – 2.028) | 0.321 | -1.949 (-6.43 – 2.531) | 0.391 |
| NYHA II | 4.98 (-2.761 – 12.721) | 0.205 | 5.034 (-3.461 – 13.528) | 0.243 |
| NYHA III | 5.018 (-2.401 – 12.438) | 0.183 | 1.068 (-7.075 – 9.21) | 0.796 |
| NYHA IV | 1.914 (-10.233 – 14.061) | 0.756 | -0.089 (-13.419 – 13.242) | 0.989 |
| LVEF 41 - 49 % | -1.174 (-6.984 – 4.637) | 0.69 | -0.837 (-7.213 – 5.54) | 0.795 |
| LVEF ≤ 40 % | -0.132 (-6.319 – 6.055) | 0.966 | 8.01 (1.22 – 14.8) | 0.021 |
| Isolated Off-Pump CABG | -1.051 (-10.945 – 8.843) | 0.834 | -1.136 (-11.993 – 9.721) | 0.836 |
| CABG combinations | 0.652 (-5.245 – 6.549) | 0.827 | 2.135 (-4.336 – 8.607) | 0.515 |
| Aortic procedures | 0.496 (-5.1 – 6.091) | 0.861 | 2.988 (-3.153 – 9.128) | 0.337 |
| POI | -7.466 (-16.539 – 1.607) | 0.106 | -2.164 (-12.121 – 7.793) | 0.668 |
| AXC | 2.628 (-5.765 – 11.022) | 0.536 | -5.232 (-14.443 – 3.979) | 0.263 |
| 1-2 transfusions | -0.8 (-5.27 – 3.67) | 0.724 | 1.232 (-3.673 – 6.137) | 0.62 |
| ≥ 3 transfusions | -2.374 (-7.92 – 3.171) | 0.398 | -2.709 (-8.794 – 3.377) | 0.38 |
| VIS 5-15 | 0.689 (-4.963 – 6.341) | 0.81 | 3.567 (-2.636 – 9.77) | 0.257 |
| VIS > 15 | 2.169 (-4.13 – 8.469) | 0.497 | 6.309 (-0.604 – 13.222) | 0.073 |
| Failure to Extubate | -1.35 (-5.266 – 2.566) | 0.496 | -0.855 (-5.153 – 3.442) | 0.694 |
| AKI | -1.968 (-6.714 – 2.778) | 0.413 | 0.697 (-4.511 – 5.905) | 0.791 |
| Pre-Frail | -6.164 (-10.305 – -2.023) | **0.004** | -2.114 (-6.658 – 2.431) | 0.359 |
| Physical frailty | -9.882 (-18.275 – -1.489) | **0.021** | -5.055 (-14.265 – 4.156) | 0.279 |
| Mental frailty | 0.796 (-7.534 – 9.126) | 0.85 | -6.726 (-15.867 – 2.415) | 0.148 |
| POD | -1.047 (-10.714 – 8.62) | 0.831 | 3.022 (-7.587 – 13.63) | 0.574 |
|  | R^2^ / R^2^ adjusted 0.353 / 0.148 | | R^2^ / R^2^ adjusted 0.222 / -0.023 | |
|  |  |  |  |  |

Supplementary Table 3: Multivariate analysis of the subgroup (CABG: coronary artery bypass graft; CI: confidence interval; LDL: low density lipoprotein; Lp(a): lipoprotein(a); LVEF: left ventricular ejection fraction; MCS: mental component summary; Mod./sev. an.: moderate/severe anemia; NYHA: New York Heart Association; PAD: peripheral arterial disease; PCS: physical component summary; POI: postoperative infection; POD: postoperative delirium; y.: years)

|  | **PCS** | | **MCS** | |
| --- | --- | --- | --- | --- |
| **Variable** | **Beta** | **BCa 95 % CI** | **Beta** | **BCa 95 % CI** |
| Female sex | -2.375 | **-3.957 – -0.791** | -0.874 | -2.725 – 0.899 |
| Age 65-74 y. | -1.286 | -2.923 – 0.246 | 3.888 | **2.097 – 5.680** |
| Age ≥ 75 y. | -4.248 | **-6.278 – -2.319** | 3.725 | **1.712 – 5.784** |
| Mild anemia | -2.409 | -4.852 – 0.012 | -0.037 | -2.632 – 2.347 |
| Mod./sev. an. | -4.939 | **-8.647 – -1.083** | 0.181 | -3.706 – 3.725 |
| Underweight | -0.015 | -4.718 – 4.623 | -1.815 | -10.892 – 5.266 |
| Overweight | -2.396 | **-4.040 – -0.676** | 0.282 | -1.566 – 2.181 |
| Obese | -4.893 | **-6.747 – -3.049** | -0.219 | -2.252 – 1.850 |
| Active smoking | -3.532 | **-5.853 – -1.250** | -1.641 | -4.183 – 0.583 |
| Former smoking | -1.884 | **-3.486 – -0.290** | -2.282 | **-4.178 – -0.505** |
| Failure to Extubate | -2.480 | -3.919 – -1.089 | -1.356 | -2.896 – 0.139 |
| AKI | -2.776 | -4.649 – -0.856 | -0.908 | -3.077 – 0.985 |
| Physical pre-frailty | -5.077 | **-8.168 – -1.684** | -0.444 | -4.349 – 3.221 |
| Physical frailty | -7.089 | **-11.563 – -2.770** | -1.746 | -6.387 – 2.819 |
|  |  |  |  |  |

Supplementary Table 4: Results of bootstrap-based resampling analysis for internal validation (AKI: acute kidney injury; BCa: Bias-Corrected and Accelerated; CI: confidence interval; MCS: mental component summary; Mod./sev. an.: moderate/severe anemia; PCS: physical component summary; y.: years)
